# USP25 Regulates the Proliferation and Apoptosis of Ovarian Granulosa Cells in Polycystic Ovary Syndrome by Modulating the PI3K/AKT Pathway *via* Deubiquitinating PTEN

**DOI:** 10.3389/fcell.2021.779718

**Published:** 2021-11-04

**Authors:** Yue Gao, Jiao Chen, Rui Ji, Jinli Ding, Yan Zhang, Jing Yang

**Affiliations:** ^1^Reproductive Medical Center, Renmin Hospital of Wuhan University & Hubei Clinic Research Center for Assisted Reproductive Technology and Embryonic Development, Wuhan, China; ^2^Department of Clinical Laboratory, Renmin Hospital of Wuhan University, Wuhan, China

**Keywords:** USP25, PTEN, PCOS, PI3K/AKT, deubiquitinating

## Abstract

**Background:** Polycystic ovarian syndrome (PCOS) is an endocrine-related disease related to abnormal folliculogenesis and is a leading cause of infertility worldwide. Inhibition of granulosa cells (GCs) proliferation and increased GCs apoptosis have been identified as the major factors in aberrant follicle maturation.

**Methods:** USP25 and PTEN expression in GCs from women with and without PCOS was analyzed using Western blotting. A PCOS-like mouse model was constructed using USP25 knockout and wild-type mice to explore the role of USP25 in PCOS. The human granular cell line KGN was cultured for proliferation and apoptosis assays, and the effect of USP25 on PTEN was investigated after transfection with shRNA-USP25 lentivirus.

**Results:** USP25 expression was found to be elevated in patients and mice with PCOS. With mouse model, we observed a reduction in PCOS symptoms in mice after USP25 deletion. Increased proliferation, reduced apoptosis, activation of the phosphoinositide-3-kinase (PI3K)/protein kinase B (AKT) signaling pathway and decreased PTEN expression were found in KGN cells after USP25 knockdown. Finally, we verified that USP25 could deubiquitinate PTEN in KGN cells.

**Conclusions:** In this study, we investigated that USP25 can regulate the PI3K/AKT signaling pathway by deubiquitinating PTEN, thus affecting the proliferation and apoptosis of GCs and contributing to the pathogenesis of PCOS.

## Introduction

In reproductive-aged women, polycystic ovarian syndrome (PCOS) is a prevalent and diverse endocrine condition. Its overall prevalence, based on Rotterdam criteria, is between 8 and 15% (Bozdag et al., 2016; Skiba et al., 2018). PCOS is often related to reproductive comorbidities, including female infertility, menstrual irregularity, ovulatory dysfunction, and pregnancy complications, as well as metabolic disorders involving insulin resistance, metabolic syndrome, and psychological risk factors, such as depression (Webber et al., 2003; Franks et al., 2008; Azziz, 2016). Despite the fact that women with PCOS have more follicles than women without the condition, none of them mature into dominant follicles (Hughesdon, 1982). Atresia, degeneration, and hypertrophy of the granulosa cell layers surrounding these follicles indicate aberrant proliferation and/or apoptosis (Broekmans and Fauser, 2006). Follicular granulosa cells (GCs) provide nutrients and growth regulators for the development of oocytes, which are required for follicular development and ovulation (Matsuda et al., 2012; Munakata et al., 2016). As a result, follicular growth can be delayed due to decreases in small antral follicle GCs proliferation and increases in GCs apoptosis, which are thought to play essential roles in the pathogenesis of PCOS (Zhao et al., 2018; Geng et al., 2021).

Ubiquitination is a post-translational modification (PTM) process in which a highly conserved ubiquitin polypeptide is added to proteins, either as a single molecule or as a chain of ubiquitin molecules, to control their stability, activity, cellular localization, protein–protein interactions, and proteolysis by the 26S proteasome (Hussain et al., 2009; Gallo and Dirks, 2016; Gao et al., 2019). The PTM of transcriptional coregulators appears to be a crucial mechanism for regulating fundamental biological functions, according to increasing data (Lecona et al., 2016; Grumati and Dikic, 2018). As a deubiquitinating enzyme, ubiquitin-specific peptidase 25 (USP25) has been reported to play a pleiotropic role in the immune response and tumorigenesis. USP25 was first identified as a negative regulator of interleukin-17 (IL-17)-mediated signaling and the inflammatory response (Zhong et al., 2012). Then, USP25 was found to regulate TNF receptor associated factor 3 (TRAF3) during toll-like receptor 4 (TLR4)-mediated signaling (Zhong et al., 2013). In addition, USP25 can stabilize tankyrases, which contribute to cancer development by enhancing Wnt/β-catenin signaling (Xu et al., 2017). However, PCOS is increasingly recognized as a proinflammatory condition. Early studies have established that circulating C-reactive protein (CRP) and inflammatory cytokines, such as interleukin-6 (IL-6) are elevated in the peripheral blood of individuals with PCOS (Benson et al., 2008), and new evidence reveals that chronic low-grade inflammation underlies the disorder’s development of metabolic abnormalities and ovarian dysfunction (González et al., 2006; Farrell and Antoni, 2010; Gonzalez, 2012). Based on the critical role of USP25 in the immune response, we speculated that USP25 might be implicated in the development of PCOS.

The enzyme phosphoinositide-3-kinase (PI3K) is essential for cell development and survival. This molecule converts membrane PI to PI-3,4,5-triphosphate, the second messenger lipid (PIP3). PIP3 binds to the membrane and recruits phosphatidylinositol-dependent kinase 1 (PDK1) and protein kinase B (AKT), which subsequently phosphorylates and activates AKT (Feng et al., 2014). Phosphatase and tensin homolog deleted on chromosome ten (PTEN) has been shown to dephosphorylate PIP3 into PI(4,5)P2(3), thus inhibiting the PI3K/AKT pathway and alleviating the biological process (Hao et al., 2011; Moon et al., 2013). It has been widely reported that the PI3K/AKT signaling pathway can primarily impact ovarian GCs. In ovarian GCs, the PI3K catalytic subunit p110d was discovered to be a crucial component of the PI3K pathway for both follicle-stimulating hormone (FSH)- and estradiol (E2)-stimulated follicular development (Li et al., 2013). Forkhead box O1 (FOXO1), a downstream target of PI3K/AKT, plays a critical role in upregulating the expression of downstream proapoptotic genes, which leads to GCs apoptosis *via* the mitochondrial pathway caused by the caspase family (Shen et al., 2012; Zhu et al., 2012; Ding et al., 2019).

For the first time, we demonstrated that USP25 expression is considerably upregulated in GCs obtained from PCOS patients. Through PCOS-like mouse models and *in vitro* experiments, we first validated that USP25 could deubiquitinate PTEN and then regulate the PI3K/AKT pathway, hence affecting the proliferation and apoptosis of GCs and leading to the development of PCOS.

## Materials and Methods

### Ethics Statement

This study was approved by the Ethics Committee of Renmin Hospital of Wuhan University. Ovarian GCs were collected from 20 PCOS patients and 20 healthy controls who underwent *in vitro* fertilization (IVF) at the Reproductive Medical Center, Renmin Hospital of Wuhan University, employing the GnRH agonist long protocol or ultralong GnRH agonist protocol. Signed informed consent was received from all subjects (No. WDRY2019-K077). The Rotterdam criteria from 2003 were used to diagnose PCOS in women, and the luteinized GCs of the patients were extracted from the follicular fluid. The follicular fluid was collected and centrifuged at 1,500 rpm for 10 min, after which the pellets were resuspended in phosphate-buffered saline (PBS). Then, GCs were separated and centrifuged at 1,800 rpm for 20 min in 50% Percoll (Biosharp, Anhui, China). The procedures for animal experiments were performed following the Guide for the Care and Use of the Animal Experiment Center Renmin Hospital of Wuhan University, and the approval ID for the animal experiment was WDRM20191203.

### Generation of a USP25-Deficient Mouse Line

By PCR amplification of px330 using primers specified in, the T7 promoter was introduced to the sgRNA template (Supplementary Table 3). Superovulated female C57BL/6 mice were mated to C57BL/6 males, and fertilized embryos were retrieved from the oviducts for mouse gene editing. Cas9 mRNA (100 ng/μl) and sgRNAs (50 ng/μl) were combined and injected into the cytoplasm of fertilized eggs with well-recognized pronuclei using a FemtoJet microinjector (Eppendorf, Hamburg, Germany) with MEM operating medium (50 ml of minimum essential medium + 0.005 g PVA). PCR analysis of DNA extracted from tail tissues was used to genotype mice (primers, Supplementary Table 4).

### Animal Model

Female prepubertal (21-day-old) wild-type mice and USP25 KO mice were randomly divided into four groups (USP25^+/+^ control, USP25^+/+^ DHEA, USP25^–/–^ control, USP25^–/–^ DHEA). Animals in the DHEA groups were injected daily with dehydroepiandrosterone (DHEA, 6 mg/100 g body weight, dissolved in 0.2 mL of sesame oil) (Abisn, Beijing, China) and given a high-fat diet (Research Diets D12492, New Brunswick, NJ, United States). Mice in the control groups were injected daily with equal amounts of sesame oil and given a normal diet. All animals were treated for 21 continuous days. Vaginal smears were taken daily at 9 a.m. from the 10th to the 20th day of treatment for at least two cycles. The estrous cycle stage was determined by a microscopic examination of the major cell types in vaginal smears following hematoxylin and eosin (HE) staining. After 21 days of continuous treatment, the mice were fasted for 12 h before the glucose tolerance test (GTT). Blood glucose detection equipment (Sinocare, Sinocare, Inc., Changsha, China) was used to measure glucose levels in tail vein blood. After fasting, fasting glucose levels were first measured. Mice were then given an intraperitoneal (IP) injection of 1.5 g/kg body weight D-glucose (Sigma, St. Louis, MO, United States), with tail samples 30, 60, 90, and 120 min afterward. Three days after the GTT experiment, the mice underwent fasting for 4 h before the insulin tolerance test (ITT) experiment. The mouse glucose levels were measured after they were fasted, and then, they were injected (IP) with 1.5 IU/kg body weight insulin (Wanbang, Jiangsu, China), with tail sampling performed 15, 30, 60, 90, and 120 min later. At the conclusion of the treatment, blood was taken from the inner canthus after the mice had fasted for 8 h. The ovaries and blood samples were taken to conduct additional research.

### Enzyme-Linked Immunosorbent Assay

The levels of testosterone (T), estradiol (E2), progesterone (P4), luteinizing hormone (LH), FSH, and insulin (INS) in mouse serum were determined by enzyme-linked immunosorbent assay kits (Xinfan Biotech, Shanghai, China) for mice. Each sample was measured in duplicate.

### Western Blotting Assay

Protein extraction and Western blot analysis were performed as described previously (Gao et al., 2019). Tissues or cultured cells were lysed in RIPA buffer (Solarbio, Beijing, China) and a protease inhibitor cocktail and phosphatase inhibitor cocktail (MedChemExpress, NJ, United States). The samples were kept on ice for 30 min and then centrifuged at 12,000 g for 25 min at 4°C. After the protein concentration was determined, protein lysates were separated by SDS-PAGE and then transferred to PVDF membranes (Millipore, MA, United States), blocked with 5% skim milk or 5% BSA in Tris-buffered saline with 0.1% Tween 20 (TBST) for 2 h at room temperature, and incubated overnight with primary antibodies. After incubation with primary antibodies, the membranes were washed in TBST three times and then incubated with their specific HRP-linked secondary antibodies (1:5,000; Jackson ImmunoResearch, PA, United States) in TBST for at least 1 h at room temperature. Bands on the membranes were visualized by ECL Western Blotting Substrate (Solarbio) using the Bio-Rad ChemiDoc^TM^ XRS + System. The related antibody information is summarized in Supplementary Table 5.

### Immunostaining

Tissues were fixed with 4% paraformaldehyde (PFA), dehydrated *via* graded ethanol solutions, and embedded in paraffin. The ovaries were sectioned longitudinally and serially at 5 mm intervals. The sections were prepared and stained with HE (Servicebio, Wuhan, China). For immunohistochemistry, the sections were subjected to a high-pressure antigen retrieval technique, and endogenous peroxidase activity was quenched. After blocking for 1 h with 0.5% bovine serum albumin, the slices were incubated with primary antibodies overnight at 4°C. The next day, the sections were incubated with HRP-conjugated secondary antibody (1:200; Jackson ImmunoResearch) at room temperature for 2 h. Immunofluorescence for colocalization was performed with cells on coverslips that were fixed with 4% PFA for 20 min at room temperature. After the cells were washed with PBS and incubated with Triton X-100/PBS for 10 min, they were blocked in 0.5% bovine serum albumin and then incubated with mouse anti-USP25 and rabbit anti-PTEN primary antibodies at 4°C overnight. The corresponding secondary antibodies were alternately applied (1:200; Jackson ImmunoResearch) for 2 h, and the nuclei were stained with 4′,6-diamidino-2-phenylindole the next day. The results were observed with a confocal microscope FV1200 (Olympus,Tokyo, Japan). The related antibody information is summarized in Supplementary Table 5.

### Cell Culture and Treatments

The human GCs line KGN was cultured in Dulbecco’s modified Eagle’s medium/nutrient mixture F-12 (DMEM/F12) (Gibco, Grand Island, NE, United States) containing 10% charcoal-stripped fetal bovine serum (Gibco) and 1% antibiotics (mixture of penicillin, streptomycin, and neomycin; Gibco) in a 37°C and 5% CO_2_ incubator (Thermo Fisher Scientific, Waltham, MA, United States). Cells were passaged every 2 days. For MG132 (100 μM, Selleck Chemicals, Houston, TX, United States) treatment, cells were harvested after 6 h of treatment. In addition, cells were treated with 100 μM cycloheximide (CHX, Selleck Chemicals, Houston, TX, United States) and collected every 12 h to assess PTEN protein stability. For inhibition of PI3K/AKT signaling, 30 μm of LY294002 (Selleck Chemicals), a specific inhibitor that blocks the PI3K/AKT pathway, was added to the cells for 12 h. Since Mg132, CHX, and LY294002 were all dissolved in DMSO, an equal volume of DMSO was added to the control cells.

### Lentiviral Particle Transduction

For knockdown of USP25 expression, KGN cells were transfected using shRNA-USP25 (GV493 vector) packaged into a lentivirus purchased from GeneChem BioTECH (Shanghai, China). The shRNA against USP25 and the negative control shRNA sequences were ACTTCTCCTGTTGACGATA and TTCTCCGAACGTGTCACGT, respectively. Infections were carried out in the presence of 5–10 mg/ml polybrene. RT-qPCR and Western blotting analysis were performed to examine the transfection efficiency.

### CCK-8 Assay

The CCK-8 assay was performed using a commercial kit (Biosharp, Anhui, China) according to the manufacturer’s protocol. Briefly, the cells were seeded on 96-well plates at a density of 5,000 cells/well. After 24, 48, and 72 h, 10 μl of reagent (CCK8) was added to the culture medium, and the plate was incubated for 2 h. The absorbance at 450 nm was detected using a microplate reader (Ensight, Perkin Elmer, Waltham, MA, United States). All CCK-8 tests were performed in triplicate and were repeated three times.

### Cell Cycle Analysis

KGN cells expressing shGV493 and shUSP25 were plated in six-well plates and starved overnight. Trypsinization was used to harvest cells after 24 and 48 h. The cells were rinsed in precooled PBS, centrifuged, and resuspended in 70% precooled ethanol (−20°C) for cell fixation before being incubated at 4°C for 24 h. The fixed cells were centrifuged and washed with PBS twice and then resuspended in 0.5 ml of propidium iodide/RNase staining buffer (BD Pharmingen, San Diego, CA, United States). A flow cytometer (BD FACSCalibur, BD Biosciences, San Diego, CA, United States) was used to analyze the cell cycle after 30 min of incubation in the dark.

### Cell Apoptosis Assay

Cells were seeded and incubated in six-well plates. A cell apoptosis assay was performed using flow cytometry. Briefly, 1 × 10^5^ cells were resuspended in 1 × binding buffer, stained with FITC-conjugated Annexin V labeling reagent (BD Pharmingen) and PI (BD Pharmingen) at RT for 15 min in the dark, and then analyzed using a flow cytometer (BD FACSCalibur, BD Biosciences) within 1 h.

### Co-Immunoprecipitation

Immunoprecipitation was performed as previously described. Proteins were extracted from the cell lysis buffer for Western blotting and IP (Beyotime, Shanghai, China). Protein extracts were subjected to centrifugation at 13,000 rpm for 10 min. Protein lysates were precleared with magnetic protein A/G beads (MedChemExpress) for 30 min before immunoprecipitation with USP25 or PTEN antibody or corresponding normal immunoglobulin G (IgG) at 4°C overnight. Then, the lysate and antibody (immunocomplex) solution were transferred to a tube containing the prewashed magnetic bead pellet. Incubate with rotation for 6 h at 4°C. The pellet was resuspended with 40 μl of 3X SDS sample buffer, briefly vortexed to mix, and briefly microcentrifuged to pellet the sample. The sample was heated to 95–100°C for 5 min and analyzed by Western blots. The related antibody information is summarized in Supplementary Figure 5.

### Quantitative Real-Time RT-PCR

TRIzol reagent (Accurate Biology, Hunan, China) was used to extract total RNA from ovaries or cultured cells. The Evo M-MLV RT Mix Kit with gDNA Clean for qPCR (Accurate Biology) was used to reverse transcribe total RNA to cDNA for qPCR. Polymerase chain reaction (PCR) was performed using a SYBR^®^ Green Premix Pro Taq HS qPCR Kit (Accurate Biology) on a CFX96 real-time PCR detection system (Bio-Rad, Hercules, CA, United States) as follows: 95°C for 30 s, followed by 40 cycles of 95°C for 5 s, 60°C for 30 s, and 65°C for 5 s. The PCR system (20 μl) comprised RNase-free H_2_O (7.2 μl), cDNA (2 μl), forward primer (0.4 μl), reverse primer (0.4 μl) and 2 × SYBR^®^ Green Pro Taq HS Premix (10 μl). All of the PCRs were performed in triplicate. Each experiment was repeated at least three times. All the primers for real-time PCR are listed in Supplementary Table 2. Data from qRT-PCR were analyzed using the 2^–△△Ct^ method.

### EDU Assay

The cells were seeded onto 24-well plates at 5.0 × 10^4^ cells/well and incubated at 37°C with 5% CO_2_ for 24 h. The culture solution was replaced by serum-free DMEM/F12 medium containing 1:1,000 EdU for a 2 h incubation. The steps were based on the directions of the EdU kit (Beyotime, Shanghai, China). Three random fields of view were observed and imaged by an inverted fluorescence microscope. Blue fluorescence indicates all cells, and red fluorescence indicates cells penetrated by EdU. The percentage of EdU-positive cells was calculated.

### Statistical Analysis

Statistical analysis was performed using the SPSS 20 program. In the figures, data from at least three independent samples are presented as the mean ± standard deviation. Student’s *t*-test was used to analyze differences in gene expression between groups. A value of *P* < 0.05 was considered significant.

## Results

### Increased Level of USP25 in PCOS Patients and Mice

**FIGURE 1:**
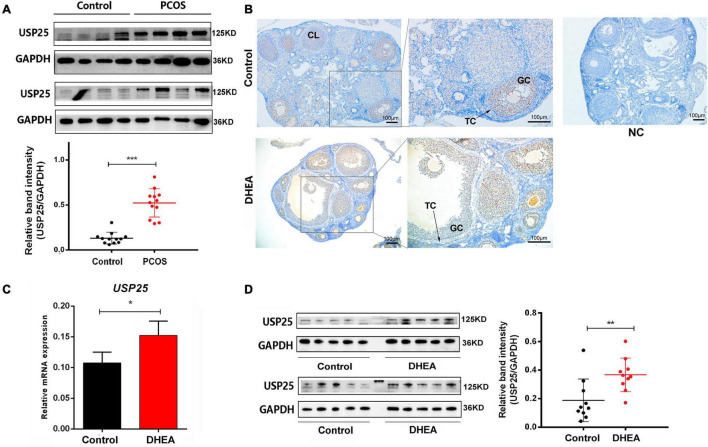
USP25 expression is upregulated in PCOS patients and mice. **(A)** USP25 protein was detected by Western blotting analysis in human luteinized GCs from patients in the control and PCOS groups (*n* = 12 per group). The protein expression level of USP25 was statistically analyzed. **(B)** Immunochemistry analysis of USP25 in the ovaries from the mice in the control and PCOS groups. Bar = 100 μm. NC, negative control. Some Positive staining of USP25 are indicated by red arrows. USP25 mRNA **(C)** and protein levels **(D)** were detected in mouse ovaries from the control and PCOS groups by quantitative real-time PCR and Western blots. The quantification of protein levels is presented on the right. In panels **(A–D)**, data are expressed as the mean ± SD. The *P*-values were calculated by an unpaired two-tailed Student’s *t*-test compared with controls. *P < 0.05, **P < 0.01, ***P < 0.001.

A total of 40 subjects (20 women with PCOS and 20 healthy women) were included in this study. The major anthropometric variables and endocrine parameters of the women are presented in Supplementary Table 1. The protein level of USP25 in the ovarian GCs of the women with PCOS was also higher than that of the healthy controls (Figure 1A). In conclusion, the expression of USP25 is higher in PCOS patients than in healthy controls.

To further explore the expression of USP25 in PCOS, we constructed a mouse PCOS model with DHEA. Mice were divided into the control and DHEA groups. We examined the localization and quantitative expression of USP25 in the mouse ovary. USP25 was mainly localized in the nucleus of GCs in mouse ovaries and was more strongly expressed in the DHEA-treated group than in the control group (Figure 1B). Similarly, both USP25 protein and mRNA levels were significantly higher in the DHEA-treated mouse ovaries than in the controls (Figures 1C,D). The above results indicated that the DHEA-treated mice have considerably higher expression of USP25 in both the ovaries and serum than the control animals.

### The Symptoms of PCOS Were Relieved in the USP25^–/–^ Mice

**FIGURE 2:**
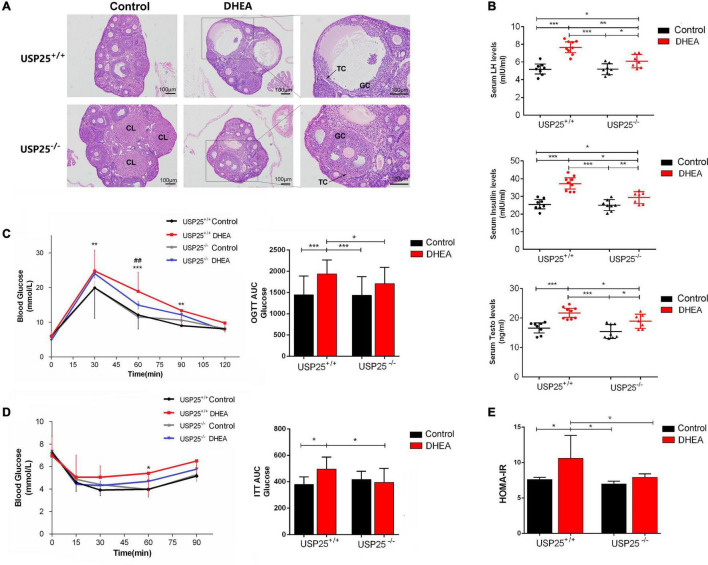
The symptoms of PCOS were relieved in the USP25^–/–^ mice. **(A)** Representative images of H&E-stained mouse ovaries for the indicated groups. Scale bar = 100 μm. TC, theca cells; GCs, granulosa cells; CL, corpus luteum. **(B)** The serum levels of LH, testosterone and insulin in mice exposed to the four treatments for 20 days (*n* = 8 per group). *P < 0.05, **P < 0.01, ***P < 0.001. **(C)** Glucose tolerance tests (GTTs) and the AUCs were assessed for the four groups. *P < 0.05 vs. USP25^+/+^; ^##^*P* < 0.01 vs. DHEA. **(D)** Insulin tolerance test (ITT) and AUCs for mice in the four groups. *P < 0.05 vs. USP25^+/+^. **(E)** Homeostasis model assessment-insulin resistance (HOMA-IR) was analyzed based on fasting blood glucose and insulin levels. Data are expressed as the mean ± SD. The *P*-values were calculated by an unpaired two-tailed Student’s *t*-test. *P < 0.05, **P < 0.01, ***P < 0.001. Four treatment groups of mice were used: USP25^+/+^ control (control), USP25^+/+^ DHEA (PCOS mice), USP25^–/–^ control (USP25 KO control mice), and USP25^–/–^ DHEA (USP25 KO PCOS mice).

To examine the potential functions of USP25 in PCOS, we generated a USP25 null mutant mouse line (Supplementary Figure 1). The PCOS model was constructed with DHEA, and the mice were divided into four groups: USP25^+/+^ control, USP25^+/+^ DHEA, USP25^–/–^ control and USP25^–/–^ DHEA. Vaginal smear detection of the estrous cycle in mice showed that the estrous cycle was almost stalled in the diestrus or metestrus phase in the mice of the USP25^+/+^ DHEA group, while the USP25^–/–^ DHEA group showed restoration of part of the cycle (Supplementary Figure 2), indicating that the estrous cycle was partially restored in the mice with PCOS after the knockout of USP25. Regarding the morphology of the ovaries, the ovaries of both the USP25^+/+^ and USP25^–/–^ control mice had follicles at various stages of development with surrounding theca cells (TC), GCs, and corpus luteum (CL), and neither structural anomalies nor ovarian cysts were discovered. When comparing the two DHEA-treated groups, the ovaries of the USP25^+/+^ DHEA group had a cyst-like appearance, considerably thinner follicular walls, and fewer granulosa cell layers than those of the DHEA-treated USP25^–/–^ mice (Figure 2A). Then, the expression of sex hormones in the serum of the four groups was determined to compare the endocrine conditions of these mice. These findings showed that the expression levels of LH and testosterone (Testo) were substantially lower in the USP25^–/–^ DHEA group than in the USP25^+/+^ DHEA group (Figure 2B). We next analyzed glucose metabolism and insulin resistance in the four groups of mice. First, the USP25^–/–^ DHEA group had drastically reduced fasting insulin levels compared with the USP25^+/+^ DHEA group (Figure 2B). Additionally, it was discovered that the areas under the curve (AUCs) of the glucose levels from the GTT and ITT analyses were substantially higher in the USP25^+/+^ DHEA group than in the USP25^–/–^ DHEA group after administration of glucose or insulin (GTT or ITT) (Figures 2C,D). Similar results were found for the homeostatic model assessment of IR (HOMA-IR) levels [defined as HOMA = fasting insulin (μUI/mL) × fasting glucose (mmol/L)/22.5] (Figure 2E). In summary, the symptoms of PCOS were significantly alleviated both endocrinologically and metabolically after USP25 was knocked out.

### The Loss of USP25 Improved the Proliferation and Inhibited the Apoptosis of KGN Cells

**FIGURE 3:**
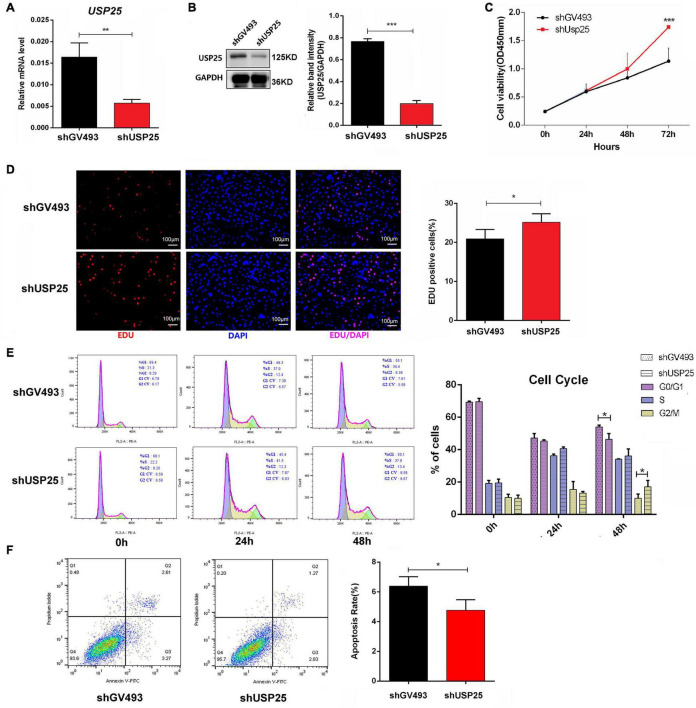
Loss of USP25 improved the proliferation and inhibited the apoptosis of human ovarian GCs. **(A)** The expression of USP25 was detected by real-time PCR after KGN cells were infected with shUSP25 or shGV493 lentivirus. **P < 0.01. **(B)** The expression of USP25 at the protein level was revealed by Western blots in the shGV493 and shUSP25 cells. The quantification of protein levels is presented on the right. Values were normalized to the GAPDH expression level and are indicated as the mean ± SD. *n* = 3. ***P < 0.001. **(C)** Cell Counting Kit-8 assay of KGN cells in the shGV493- and shUAP25-treated cells. ***P < 0.001. **(D)** Representative images and the quantification of EdU (+) in KGN cells. Scale bar = 100 μm. *P < 0.05. **(E)** Effects of USP25 knockdown on KGN cell cycle distribution determined using flow cytometry. *P < 0.05 for each group. **(F)** Apoptosis of KGN cells in each group was detected by flow cytometry. A cartogram of apoptosis of KGN cells is shown on the right. *P < 0.05. Data are expressed as the mean ± SD. The *P*-values were calculated by an unpaired two-tailed Student’s *t*-test.

To better understand the functions of USP25 in ovarian GCs, we stably downregulated USP25 expression in KGN cells using a lentiviral system (Figure 3A). Both USP25 mRNA and protein levels were significantly reduced in the shUSP25 cells, as shown in Figures 3A,B. Experiments with the Cell Counting Kit-8 (CCK-8) and a 5-ethynyl-20-deoxyuridine (EdU) assay illustrated that the proliferation of KGN cells in the shUSP25 group was augmented compared with that in the shGV493 group (Figures 3C,D). We next explored the cell cycle distribution in the two groups of cells. Flow cytometry revealed that the shUSP25 cells had considerably fewer cells in the G0/G1 phase and more cells in the G2/M phase than the controls (Figure 3E). For the study of cell apoptosis, annexin V/propidium iodide (PI) flow cytometry indicated that the apoptotic rate was remarkably reduced after USP25 was knocked down (Figure 3F). Collectively, these data indicate that the loss of USP25 improves the proliferation and inhibits the apoptosis of KGN cells.

### USP25 Enhanced the Proliferation and Apoptosis of KGN Cells Through the PI3K/AKT Signaling Pathway

**FIGURE 4:**
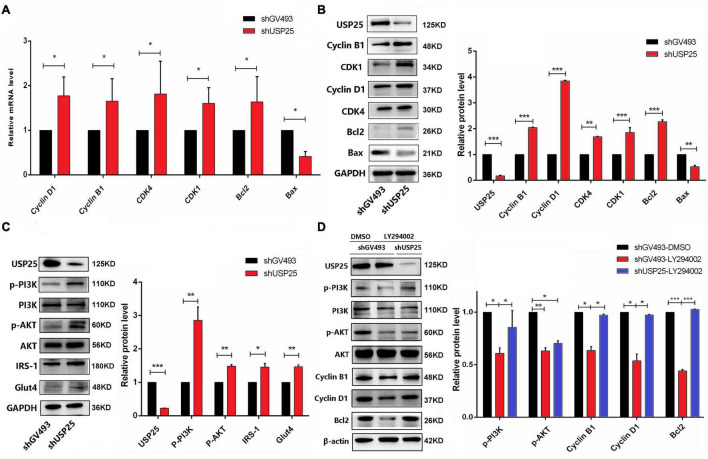
USP25 regulated the proliferation and apoptosis of human ovarian GCs through the PI3K/AKT signaling pathway. **(A)** The expression of Cyclin D1, Cyclin B1, CDK4, CDK1, Bcl2, and Bax at the mRNA level. **(B)** Western blotting for USP25, Cyclin B1, CDK1, Cyclin D1, CDK4, Bcl2, and Bax analysis. Relative expression levels were normalized to that of GAPDH. **(C)** Western blot assays were conducted to detect the PI3K/AKT pathway and related proteins (USP25, PI3K, AKT, IRS-1, and Glut4). Relative p-PI3K and p-AKT expression levels were normalized to those of PI3K and AKT. The expression of USP25 and Glut4 was normalized to that of GAPDH. **(D)** Expression of PI3K/AKT pathway-related proteins after treatment with the specific inhibitor LY294002. Relative expression levels were normalized to that of β-actin. Relative p-PI3K and p-AKT expression levels were normalized to those of PI3K and AKT. In panels **(A–D)**, the data represents the mean ± SD. *P < 0.05, **P < 0.01, ***P < 0.001.

Since the cell cycle distribution showed significant changes in G0/G1 and G2/M phase cells after USP25 knockdown, we examined the expression of some molecules that are associated with the regulation of G0/G1 and G2/M phases. As shown in Figures 4A,B, Cyclin D1 and cyclin-dependent kinases 4 (CDK4), which govern the G0/G1 phase, and Cyclin B1 and cyclin-dependent kinases 1 (CDK1), which regulate the G2/M phase, were all considerably increased in the shUSP25 cells at the mRNA and protein levels. Additionally, the antiapoptotic gene *B-cell lymphoma-2* (*Bcl2*) was increased while the proapoptotic gene *Bcl2-associated X* (*Bax*) was decreased after USP25 was knocked down in KGN cells (Figures 4A,B).

In view of the important role of USP25 in inflammation, we investigated the changes in some critical inflammation-associated proteins, such as IL-6, p-P65, p-P38, and p-ERK1/2, in the shGV493 and shUSP25 cells (Supplementary Figure 4). However, the results were contrary to our human and mouse phenotype, implying that USP25 might not have a role in the development of PCOS by modulating the inflammatory response. The PI3K/AKT signaling pathway is well-known for its importance in cell proliferation, apoptosis, glucose metabolism, and a variety of other physiological processes. Thus, the changes in the PI3K/AKT pathway were next investigated. In comparison to those in the control group, PI3K/AKT pathway-related proteins were significantly activated in KGN cells after USP25 knockdown (Figure 4C). Moreover, the protein levels of glucose transporter proteins glucose transporter 4 (GLUT4) and insulin receptor substrate 1 (IRS-1) were increased, indicating that the glucose transport ability of the shUSP25 group was improved (Figure 4C).

To further confirm that USP25 affected the proliferation and apoptosis of KGN cells *via* the PI3K/AKT pathway, we blocked the pathway using the specific inhibitor LY294002. When LY294002 was added, the protein expression levels of Cyclin B1, Cyclin D1 and Bcl2 were significantly downregulated compared to those of the DMSO control group in shGV493 cells (Figure 4D), indicating that PI3K/AKT inhibition could weaken proliferation and increase apoptosis in KGN cells. Compared to that of the shGV493 cells with LY294002, the expression of p-PI3K, Cyclin D1, Cyclin B1 and Bcl2 was significantly upregulated in the shUSP25 cells with the same inhibitor, and there was no difference in the expression in the shGV493 group with DMSO (Figure 4D). Therefore, knocking down USP25 partially reversed the effects of the specific inhibitor. In conclusion, the downregulation of USP25 expression can activate the PI3K/AKT signaling pathway, thereby promoting the proliferation and glucose transport of KGN cells while inhibiting apoptosis.

### PTEN Is Involved in USP25-Mediated Regulation of Granulosa Cells in Polycystic Ovarian Syndrome

**FIGURE 5:**
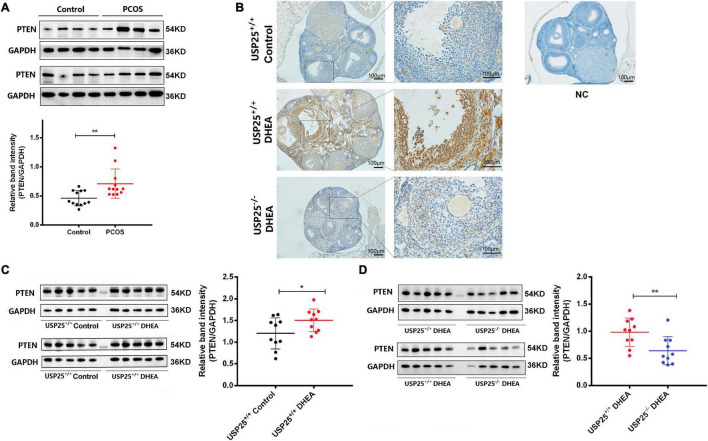
The expression of PTEN in women and mice with PCOS. **(A)** PTEN protein was detected by Western blotting analysis in human luteinized GCs from patients in the control and PCOS groups (*n* = 12 per group). The protein expression level of PTEN was statistically analyzed on the right. **(B)** Immunochemistry analysis of PTEN in the ovaries from the USP25^+/+^control, USP25^+/+^ DHEA, and USP25^–/–^ DHEA groups. Bar = 100 μm. NC, negative control. Some Positive staining of PTEN are indicated by red arrows. PTEN protein levels **(C,D)** were detected in the mouse ovaries from the four groups by Western blots. The quantification of protein levels is presented on the right. Data are expressed as the mean ± SD. The *P*-values were calculated by an unpaired two-tailed Student’s *t*-test compared with the controls. *P < 0.05, **P < 0.01.

Generally, PTEN indirectly inhibits the phosphorylation of AKT, and its activation or inactivation results in decreased or increased AKT activity (Iwase et al., 2009). Given the strong relationship between PTEN and the PI3K/AKT pathway, we wondered whether PTEN was also involved in the regulation of the PI3K/AKT pathway in ovarian GCs in PCOS. The expression of PTEN in the ovaries of the PCOS patients and the healthy controls was first compared. As demonstrated in Figure 5A, PTEN expression was considerably higher in the GCs of the PCOS patients than in the healthy individuals. With the PCOS mouse model, we examined whether the PTEN protein was mainly localized in the nucleus and cytoplasm of ovarian GCs (Figure 5B). PTEN expression was significantly upregulated after DHEA treatment compared to that in the controls and was more pronounced in the GCs of cyst-like altered follicles. In contrast, when USP25 was knocked out, PTEN expression in the ovaries of the mice with PCOS was significantly diminished (Figure 5B). Furthermore, in the mouse ovaries, PTEN expression was substantially higher in the DHEA-treated WT mice than in the controls (Figure 5C). In a comparison of the two DHEA-treated groups, the expression of PTEN was considerably lower in the USP25 KO mice than in the WT mice (Figure 5D), demonstrating that USP25 may influence the pathogenesis of PCOS *via* PTEN.

### USP25 Could Deubiquitinate PTEN in KGN Cells

**FIGURE 6:**
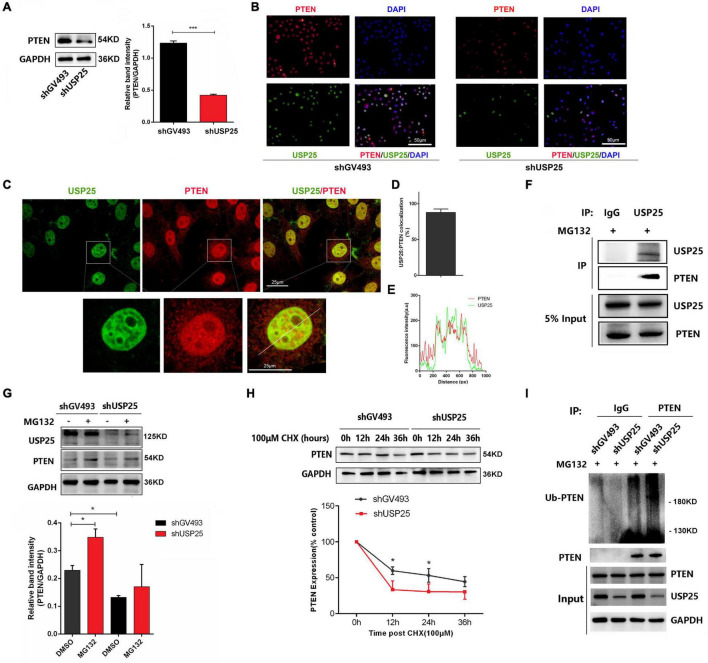
USP25 could deubiquitinate PTEN in KGN cells. PTEN protein **(A)** expression in the shGV493 and shUSP25 cells. **(B)** Expression of USP25 and PTEN was detected by immunocytochemical staining in shGV493 and shUSP25 KGN cells. Bar = 50 μm. **(C–E)** Colocalization of USP25 with PTEN was quantified. **(E)** Line scans of a cell costained against USP25 and PTEN at the position shown by the white line in the zoomed images in panel **(C)**. Scale bar = 25 μm. **(F)** Interaction between USP25 and PTEN in KGN cells determined by coimmunoprecipitation analysis using rabbit IgG or anti-USP25 antibodies. **(G)** PTEN protein degradation was observed after 6 h of exposure to 100 μM proteasome inhibitor MG132. **(H)** Stabilization of PTEN protein expression following treatment with 100 μm cycloheximide (CHX) for the indicated times. The level of GAPDH was used as an internal control for total protein. PTEN levels were quantified and are expressed as percentages relative to the control. **(I)** Immunoprecipitation assay of PTEN ubiquitination in KGN cells. KGN cells were transfected with USP25 or GV493 lentiviral vectors and treated with 100 μM MG132 for 6 h. Whole-cell lysates were prepared and subjected to immunoprecipitation using anti-PTEN antibodies. Immunoprecipitates were probed to detect the polyubiquitination of PTEN using anti-ubiquitin antibodies. Approximately 5% of the cell lysates used for coimmunoprecipitation were loaded as the inputs. Immunoglobulin G (IgG) was used as a control. Data are expressed as the mean ± SD. The *P*-values were calculated by an unpaired two-tailed Student’s *t*-test compared with the controls. *P < 0.05, ***P < 0.001.

To further investigate the interaction between PTEN and USP25, we examined PTEN expression in the shGV493 and shUSP25 KGN cells. The results revealed that when USP25 was knocked down, the protein levels of PTEN were downregulated (Figures 6A,B). Immunofluorescence images of KGN cells illustrated that PTEN colocalized with USP25 in the nucleus (Figures 6C–E). Then, coimmunoprecipitation experiments indicated that USP25 could interact with PTEN in KGN cells (Figure 6F). Overall, these results suggest that USP25 could interact with PTEN in KGN cells.

To further examine the role of USP25 in regulating PTEN, we tested whether USP25 could directly deubiquitinate PTEN and thereby increase the PTEN protein level. We treated KGN cells with MG132 to block the degradation of ubiquitinated proteins by proteasomes and found that USP25-induced downregulation of PTEN expression could be blocked by the addition of MG132 (Figure 6G). Moreover, the PTEN half-life was dramatically reduced in the cells with downregulated USP25 expression, according to protein stability analyses (Figure 6H). These findings revealed that USP25 was involved in proteasome-mediated PTEN degradation. To further verify our hypothesis, we used coimmunoprecipitation assays to examine the ubiquitination of PTEN in KGN cells, and the results showed that decreased USP25 expression resulted in increased PTEN ubiquitination (Figure 6I). Taken together, these findings indicate that PTEN protein levels are regulated by USP25-mediated deubiquitination in KGN cells.

## Discussion

**FIGURE 7:**
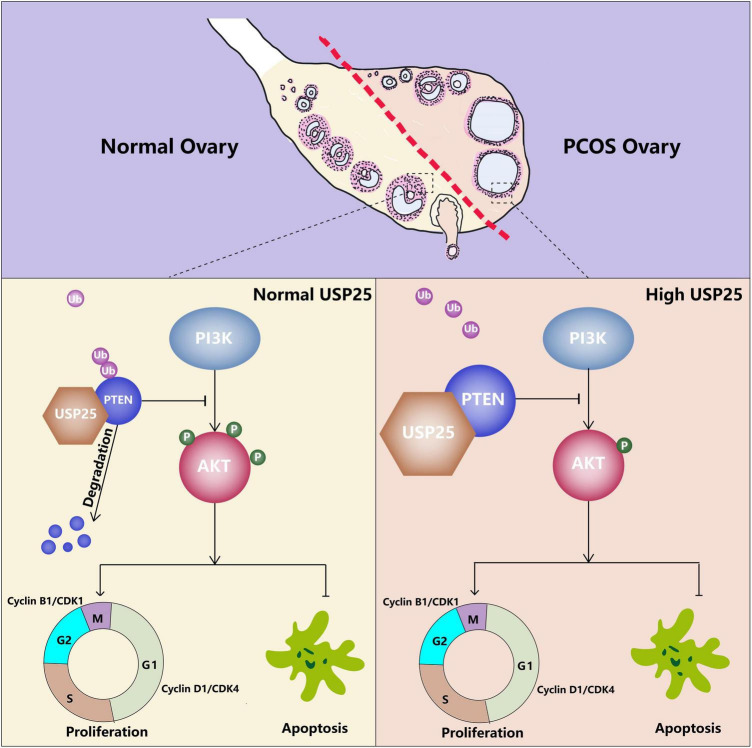
Proposed model of the effect of USP25 during PCOS pathogenesis. Upregulated USP25 expression in the GCs of women with PCOS inhibits the ubiquitination of PTEN. Increased expression of PTEN negatively regulates the PI3K/AKT signaling pathway, thereby inhibiting granulosa cell proliferation and promoting granulosa cell apoptosis, leading to the development of PCOS.

For the first time, the importance of the deubiquitinating enzyme USP25 in the pathogenesis of PCOS was revealed in this study. We discovered that USP25, as a deubiquitinating enzyme, could deubiquitinate PTEN, thereby negatively affecting the PI3K/AKT signaling pathway, leading to decreased proliferation and increased apoptosis in KGN cells, resulting in PCOS (Figure 7).

Using a mouse PCOS model, we found that both the endocrine and metabolic conditions of the mice with PCOS were improved when USP25 was knocked out. For the endocrine status, the KO mice showed restoration of part of their estrous cycle, and LH and T levels in the serum declined considerably. In terms of the metabolic condition, GTT and ITT results, as well as HOMA-IR levels in mice, all revealed that the deletion of USP25 alleviated the symptoms of insulin resistance in the animals with PCOS. The PI3K/AKT signaling pathway is the main pathway of insulin signal transduction and was significantly enhanced in the USP25 knockdown KGN cells. In addition, the expression of GLUT4 and IRS-1, major mediators of glucose removal from the circulation and a key regulator of whole-body glucose homeostasis (Huang and Czech, 2007), was dramatically elevated after USP25 was knocked down in KGN cells. Insulin resistance and hyperandrogenemia, major complications of PCOS, were both alleviated in the USP25-deficient mice with PCOS, indicating the promise of USP25 in the diagnosis and treatment of PCOS in the future.

As a deubiquitinating enzyme, USP25 has been associated with a multitude of signaling pathways, including the IL-17-mediated inflammatory response (Zhong et al., 2012), virus- and pathogen-induced innate immune responses (Lin et al., 2015), and Wnt signaling (Xu et al., 2017). In contrast, the relationship between the inflammatory response and the development of PCOS has also been well-documented. At first, IL-6 and tumor necrosis factor α (TNF-α) expression is low but significantly increased in circulation of women with PCOS (Ghowsi et al., 2018). Second, decreased expression of phosphorylated extracellular signal regulated kinase (ERK) 1/2 and increased expression of NF-κB p65 were found in women with PCOS (Nelson-Degrave et al., 2005; Lan et al., 2015; Gu et al., 2016). Finally, the activity of p-p38 MAPK was found to be increased in the GCs and cumulus-oocyte complexes (COCs) of the dihydrotestosterone (DHT)-treated mouse ovaries (Jin et al., 2020). To determine whether USP25 regulates the pathogenesis of PCOS by modulating the inflammatory response, we examined the expression of the relevant inflammatory pathways in the USP25 knockdown and control KGN cells. USP25 knockdown was followed by increased expression of IL-6, p-P38, and p-P65 and decreased expression of p-ERK1/2 in KGN cells, which is contrary to our human and mouse phenotype. Therefore, we reviewed the literature and performed experiments, and eventually, we revealed the role of the PI3K/AKT signaling pathway in USP25-mediated PCOS pathogenesis.

It has been widely reported that the PI3K/AKT signaling pathway plays a role in ovarian follicle development (Li et al., 2017). AKT’s activities in increasing proliferation are mediated by the cell cycle regulators Cyclin D1 and Cyclin B1 (Liu et al., 2020; Jiang et al., 2021). As downstream factors of the PI3K/AKT signaling pathway, the antiapoptotic gene *Bcl2* and the proapoptotic gene *Bax* play crucial roles in the regulation of GCs apoptosis during follicular development (Hu et al., 2004; John et al., 2009; Gong et al., 2020). We found that after USP25 was knocked down, the PI3K/AKT signaling pathway was activated in KGN cells. The pathway-related molecules involved in cell proliferation and apoptosis also changed in response to the change in the pathway. These results indicate that USP25 can regulate the proliferation and apoptosis of KGN cells through the PI3K/AKT signaling pathway. This conclusion was further confirmed by the application of the PI3K/AKT signaling-specific inhibitor LY294002.

PTEN, a well-known negative regulator of the PI3K/AKT pathway, has been repeatedly investigated as a tumor suppressor gene. The importance of PTEN in PCOS has been increasingly revealed in recent years. PTEN levels in GCs were reported to be higher in PCOS patients and were linked to insulin concentrations in follicular fluid (Iwase et al., 2009). In the primordial follicle, PTEN has a role in initiation, development, apoptosis, and atresia (Ouyang et al., 2013). In our study, PTEN expression was considerably upregulated in the women and mice with PCOS, which is consistent with previous reports. PTEN can be ubiquitinated by a variety of ubiquitinating enzymes. Tripartite motif containing 25 (TRIM25) can polyubiquitinate PTEN and activate the AKT/mTOR pathway in non-small cell lung cancer (He et al., 2021). Smad ubiquitination regulatory factor 1 (Smurf1) can mediate PTEN ubiquitylation to promote PTEN wild-type glioblastoma growth (Xia et al., 2020). In this study, we discovered that USP25, a deubiquitinating enzyme, can deubiquitinate PTEN in ovarian GCs, thereby regulating the PI3K/AKT signaling pathway in the cells, leading to the pathogenesis of PCOS.

## Conclusion

In conclusion, we have revealed the critical role of the deubiquitinating enzyme USP25 in PCOS pathogenesis. We expect to provide a theoretical basis for the diagnosis and therapy of PCOS in the future.

## Data Availability Statement

The original contributions presented in the study are included in the article/Supplementary Material, further inquiries can be directed to the corresponding authors.

## Ethics Statement

The studies involving human participants were reviewed and approved by the Ethics Committee of the Renmin Hospital of Wuhan University. The patients/participants provided their written informed consent to participate in this study. The animal study was reviewed and approved by the Animal Experiment Center, Renmin Hospital of Wuhan University.

## Author Contributions

YG performed the experiments and wrote the manuscript. JC and YZ designed the experiments. JD provided the experimental materials and reagents, and gave professional advices. RJ collected the mouse samples and gave experimental guidance. JY organized the whole project. All authors permitted and approved the submitted manuscript.

## Conflict of Interest

The authors declare that the research was conducted in the absence of any commercial or financial relationships that could be construed as a potential conflict of interest.

## Publisher’s Note

All claims expressed in this article are solely those of the authors and do not necessarily represent those of their affiliated organizations, or those of the publisher, the editors and the reviewers. Any product that may be evaluated in this article, or claim that may be made by its manufacturer, is not guaranteed or endorsed by the publisher.
