# Supplementary material for: USP25 Regulates the Proliferation and Apoptosis of Ovarian Granulosa Cells in Polycystic Ovary Syndrome by Modulating the PI3K/AKT Pathway via Deubiquitinating PTEN
Source: Front Cell Dev Biol. 2021 Nov 4;9:779718. doi: 10.3389/fcell.2021.779718 (PMC8599287; doi:10.3389/fcell.2021.779718)
Supplement: Supplementary file 1 [file Data_Sheet_1.DOCX]

**Supplemental Information**

**USP25 Regulates Proliferation and Apoptosis of Ovarian Granulosa Cells in Polycystic Ovary Syndrome by Modulating PI3K/AKT Pathway via Deubiquitinating PTEN**

Yue Gao, Jiao Chen, Rui Ji, Jinli Ding, Yan Zhang, Jing Yang


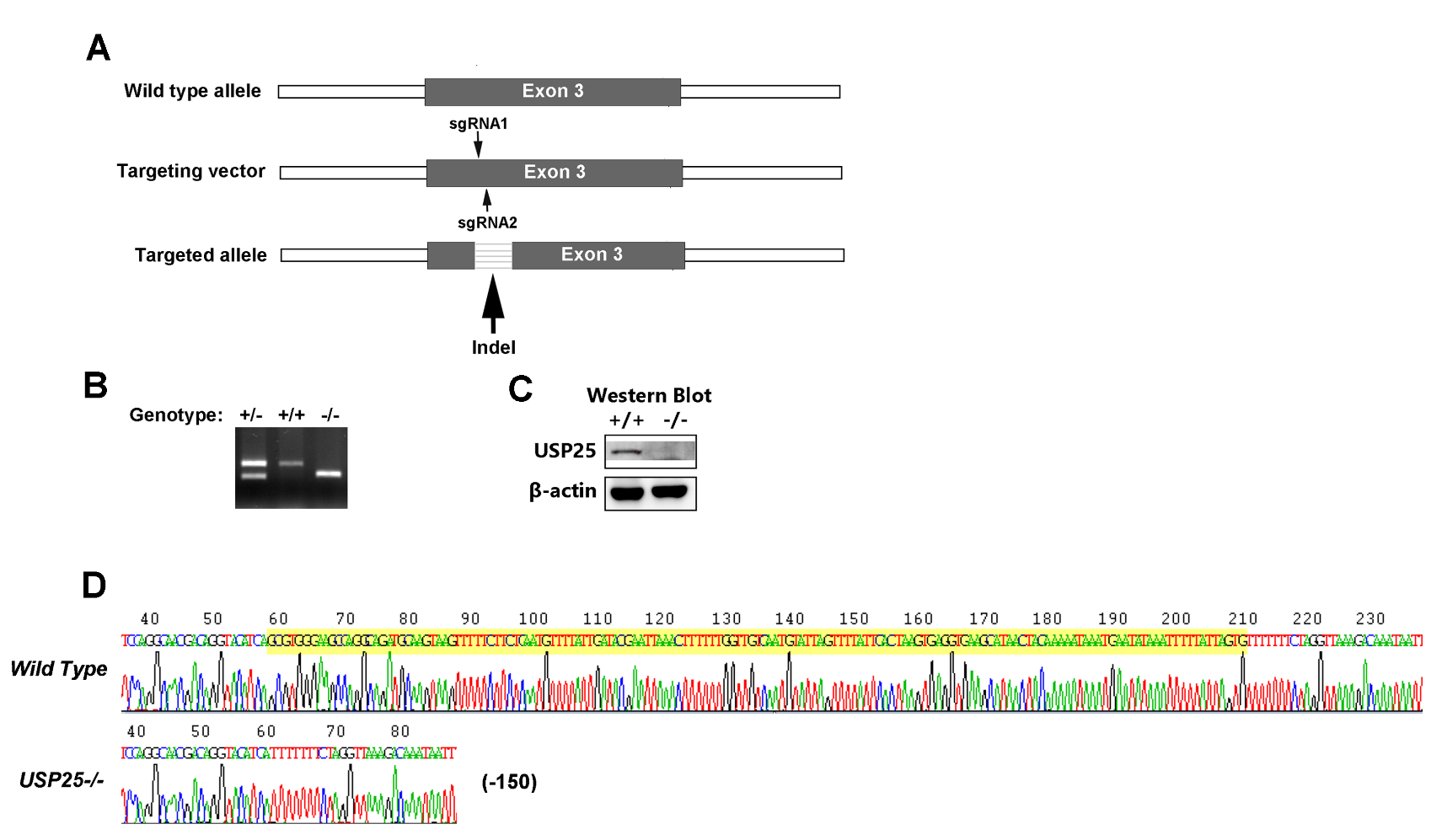
**Supplemental Figures and legends**

**Supplementary Figure 1. Generation of *USP25*^-/-^ mice.** (A) Schematic diagram of sgRNA-targeting sites at USP25 locus and experimental design. (B) Genotyping by PCR showed a 434-bp band for wild-type allele and a 284-bp band for mutant allele, simultaneously. (C) The USP25 protein is absent in USP25 knockout ovary. (D) PCR-Seq assay of editing events in *USP25* target. The gene structure of *USP25* with its target sequences is shown above, and analysis of the mutated target genes by Sanger sequencing is shown below. The nucleotide painted yellow represents the deleted nucleotide. The number after‘-’represents the number of deleted bases.


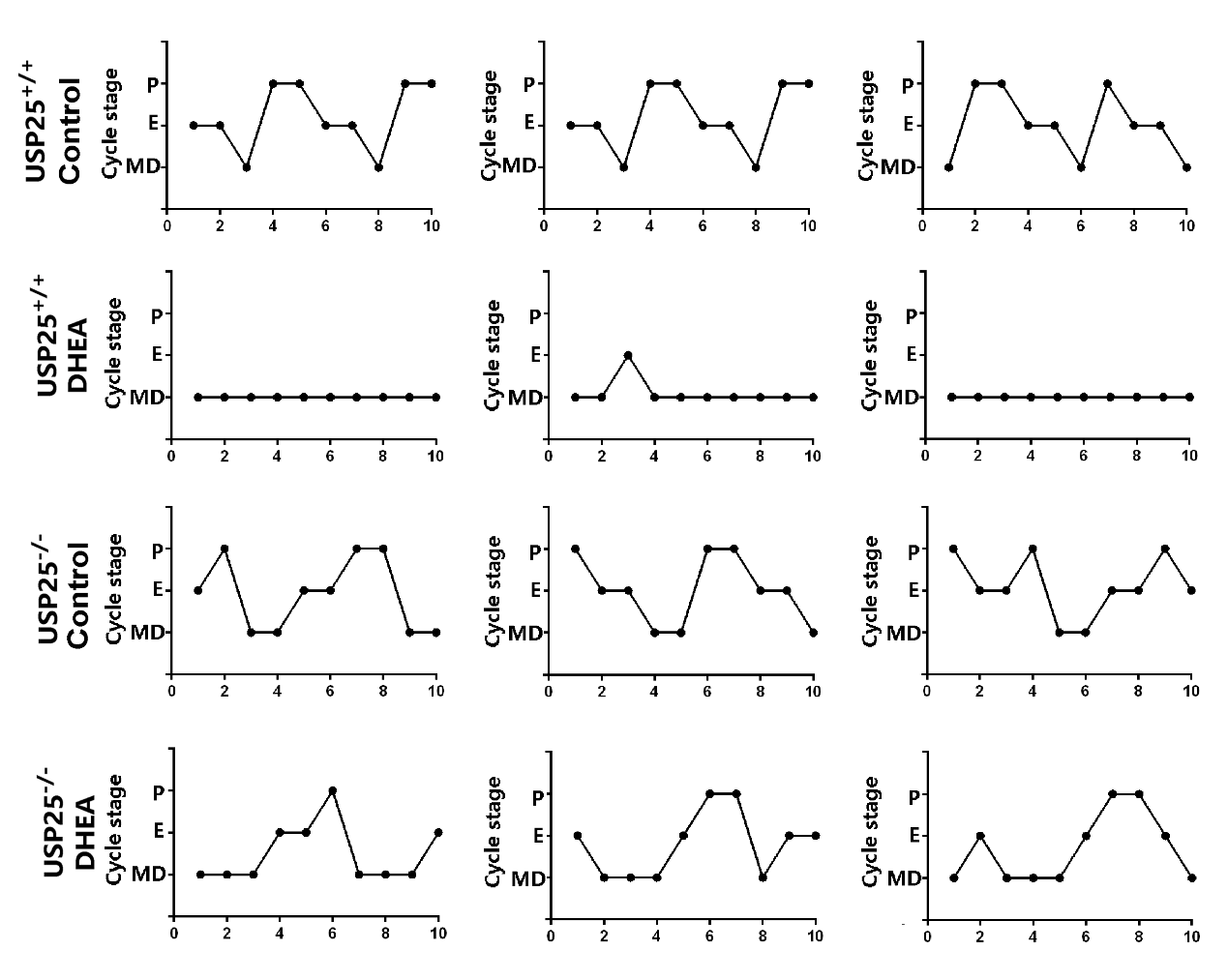


**Supplementary Figure 2.** Representative estrous cycles of three mice from each group. D, diestrus; M, metestrus; E, estrus; P, proestrus.


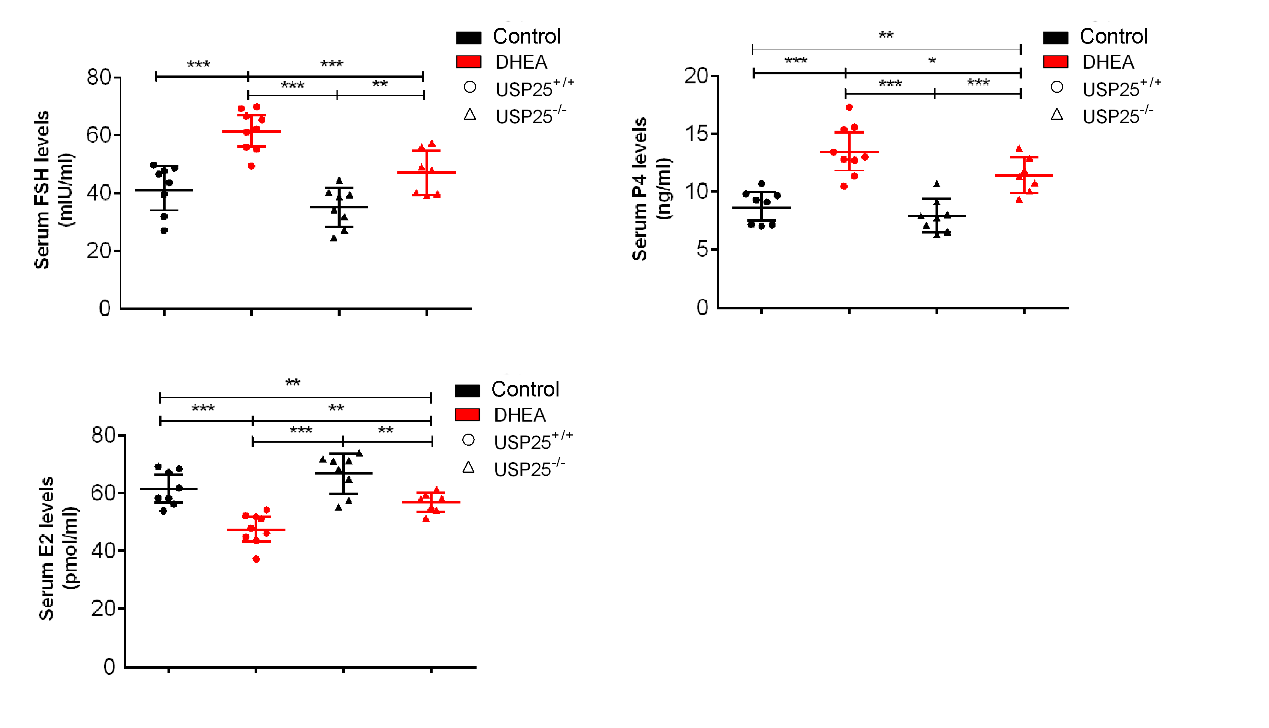


**Supplementary Figure 3.** The serum levels of estradiol (E2), progesterone (P4) and follicle-stimulating hormone (FSH) of mice exposed to the four groups for 20 days (n = 8 per group). Data are expressed as the mean±SD. The P values were calculated by an unpaired two-tailed Student’s t-test. **P＜0.01，***P＜0.001.


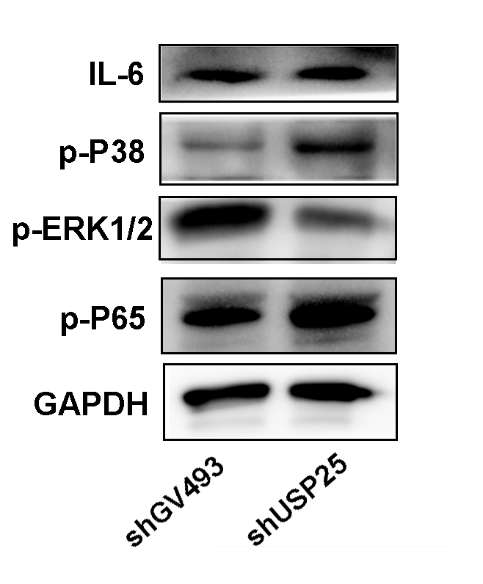


**Supplementary Figure 4.** The protein levels of IL-6, p-P38, p-ERK1/2 and p-p65 in shGV493 and shUSP25 treated KGN cells.

**Supplemental Tables**

**Supplemental Table 1.** Clinical and biochemical indicators of women with and without PCOS involved in Elisa and Western blot

|  | **PCOS**  **(n=20)** | **Control**  **(n=20)** | ***P* Value** |
| --- | --- | --- | --- |
| **Age (years)** | 29.17±3.94 | 29.30±3.28 | 0.903 |
| **BMI (kg/m^2^)** | 24.81±4.18 | 23.46±8.30 | 0.488 |
| **Basal FSH (mIU/ml)** | 6.35±1.97 | 7.31±1.25 | 0.054 |
| **Basal LH (mIU/ml)** | 7.65±5.84 | 4.66±2.19 | 0.029 |
| **LH/FSH** | 1.20±0.74 | 0.64±0.30 | 0.003 |
| **AMH (ng/ml)** | 8.57±3.98 | 3.55±1.39 | <0.0001 |
| **E2 (pg/ml)** | 48.37±13.75 | 47.69±14.42 | 0.870 |
| **P4 (ng/ml)** | 0.66±0.39 | 0.90±1.17 | 0.374 |
| **Fasting blood-glucose (mmol/l)** | 5.04±0.57 | 4.90±0.49 | 0.367 |

Data are presented as mean ± SD, BMI: Body mass index, LH: luteinizing hormone, FSH: follicle stimulating hormone, E2: estrogen P4: progestogen, AMH: anti-Müllerian hormone.

**Supplemental Table 2.** Primers used for quantitative RT-PCR.

| Gene | Sequence of Forward Primer  (5’→3’) | Sequence of Reverse Primer  (5’→3’) |
| --- | --- | --- |
| Human | | |
| USP25 | CGGTCCCAAACGATTCCC | CTCCCTGTTCTGTTGTGCT |
| PTEN | CGTTACCTGTGTGTGGTGATA | CTCTGGTCCTGGTATGAAGAATG |
| Cyclin D1 | CGCAAACACGCGCAGACCTT | ACAGGAAGTTGTTGGGGCTC |
| Cyclin B1 | TCTGGATAATGGTGAATGGA | GATGTGGCATACTTGTTCTT |
| CDK4 | CAGCTACCAGATGGCACTTAC | GATACAGCCAACACTCCACAT |
| CDK1 | GGCCAGAAGTGGAATCTTTA | TTCGTTTGGCTGGATCATAG |
| Bcl2 | GGATGCCTTTGTGGAACTGT | CACTTGTGGCTCAGATAGGC |
| Bax | CACTGAAGCGACTGATGTC | TCAGCCCATCTTCTTCCA |
| IGF1 | AACAAGCCCACAGGGTATG | ACATCTCCAGCCTCCTTAGA |
| IGF1R | AAGGCTGTGACCCTCACCAT | CGATGCTGAAAGAACGTCCAA |
| GAPDH | AGAAGGCTGGGGCTCATTTG | AGGGGCCATCCACAGTCTTC |
| Mouse | | |
| GAPDH | GGTGAAGGTCGGTGTGAACG | CTCGCTCCTGGAAGATGGTG |
| USP25 | GGACTCTTTCGGTGGTTATC | CAAGTCTGGAGGTAAGGTTTC |
| PTEN | CAGTAGAGGAGCCATCAAATC | GAGTCAGTGGTGTCAGAATATC |

**Supplemental Table 3.** sgRNA sequences and primers sgRNA sequences

|  | Sequence (5’-3’) |
| --- | --- |
| USP25-sgRNA1 | CAACGACAGGTACATCAGCG |
| USP25-shRNA2 | AACGACAGGTACATCAGCGT |

**Supplemental Table 4.** Primer used for genotyping

|  | Sequence (5’-3’) | Products (bp) |
| --- | --- | --- |
| USP25-F | GCCTTCCTCACTGCAAAGAATG | 434 (wt)  284 (del) |
| USP25-R | AGTCTCAACGCAATACTCAGCT |  |

**Supplemental Table 5.** Primary antibodies used in this study.

| Peptide/ protein target | Host | Dilution used | Manufacturer |
| --- | --- | --- | --- |
| GAPDH | Rabbit | 1:3000 | Proteintech Group Inc |
| USP25 | Rabbit | 1:200 for IHC and IF  1:1000 for Western blot | Abcam |
| USP25 (For IP) | Rabbit | 2.5 μg | Proteintech Group Inc |
| PTEN | Mouse | 1:100 for IHC  1:200 for IF  1:1000 for Western blot | Proteintech Group Inc |
| PTEN (For IP) | Rabbit | 1:50 | Cell Signaling Technology |
| Cyclin D1 | Rabbit | 1:1000 | Proteintech Group Inc |
| Cyclin B1 | Rabbit | 1:1000 | Proteintech Group Inc |
| CDK4 | Rabbit | 1:1000 | Proteintech Group Inc |
| CDK1 | Rabbit | 1:1000 | Proteintech Group Inc |
| p-PI3K | Rabbit | 1:1000 | Cell Signaling Technology |
| PI3K | Mouse | 1:1000 | Cell Signaling Technology |
| p-AKT | Rabbit | 1:1000 | Cell Signaling Technology |
| AKT | Rabbit | 1:1000 | Cell Signaling Technology |
| Glut4 | Mouse | 1:1000 | Proteintech Group Inc |
| Ubiquitin | Mouse | 1:1000 | Cell Signaling Technology |
| β-actin | Mouse | 1:3000 | Sigma |
| Bcl2 | Rabbit | 1:1000 | Proteintech Group Inc |
| Bax | Rabbit | 1:1000 | Proteintech Group Inc |
