# Supplementary figures and images for: USP25 Regulates the Proliferation and Apoptosis of Ovarian Granulosa Cells in Polycystic Ovary Syndrome by Modulating the PI3K/AKT Pathway via Deubiquitinating PTEN
Source: Front Cell Dev Biol. 2021 Nov 4;9:779718. doi: 10.3389/fcell.2021.779718 (PMC8599287; doi:10.3389/fcell.2021.779718)

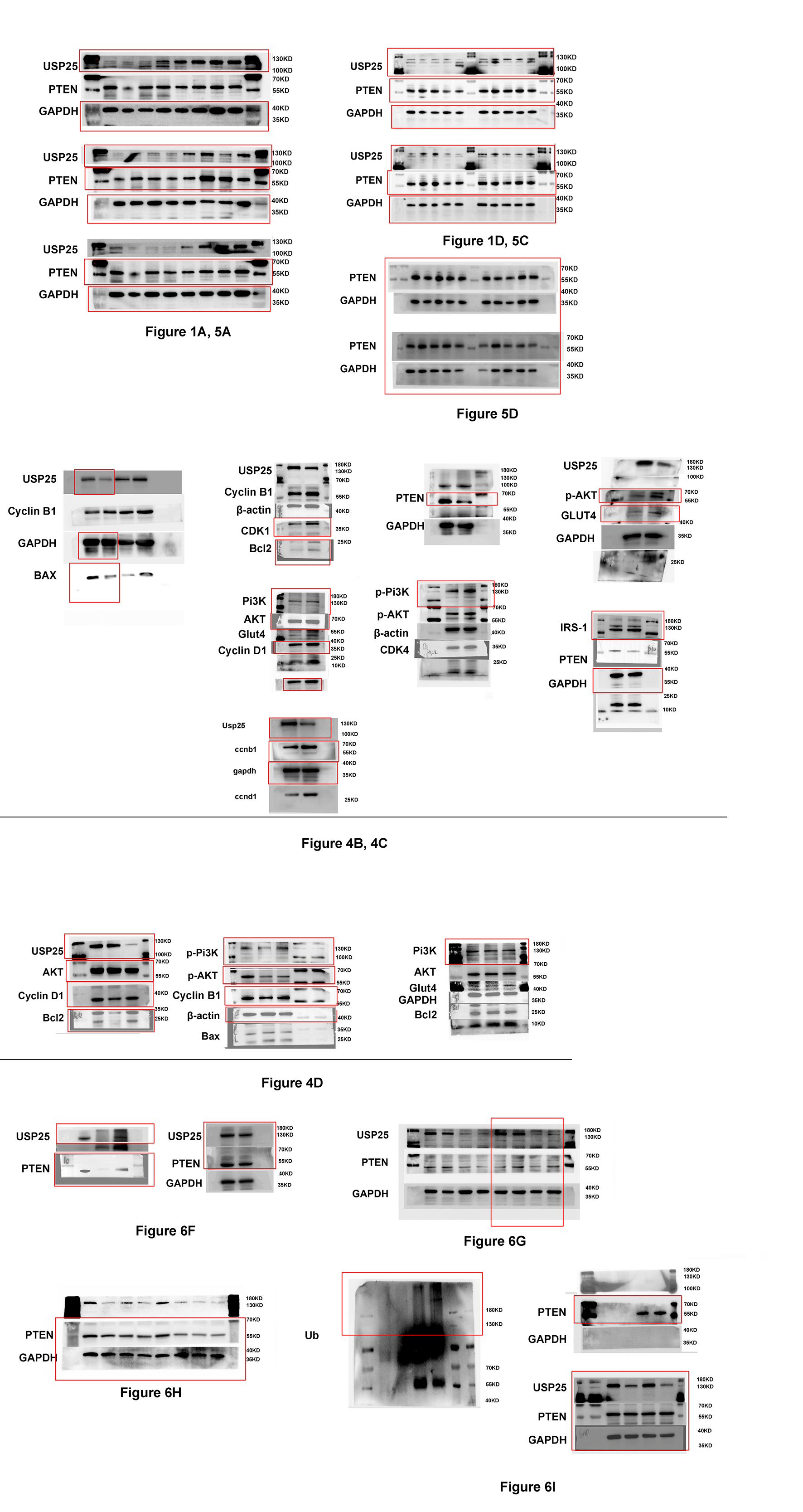

Supplement: Supplementary file 2 [file Image_1.TIF]
